# The Peels of Fruits and Vegetables: An Increasingly Recognized Source of Bioactive Compounds for Biomedical Applications

**DOI:** 10.3390/plants15070991

**Published:** 2026-03-24

**Authors:** Juan Manuel Favela-Hernández, Lucia Delgadillo-Ruiz, Gloria G. Guerrero-Manriquez

**Affiliations:** 1 Facultad de Ciencias Químicas, Campus Gómez Palacio, Universidad Juárez Del Edo de Durango, Gómez Palacio 35019, Dgo., Mexico; juan.favela@ujed.mx; 2 Unidad Académica de Ciencias Biológicas, Universidad Autónoma de Zacatecas, “Francisco García Salinas”, Zacatecas 98066, Zac., Mexico; luciadelgadillo@uaz.edu.mx

**Keywords:** bio-waste, by-products, peels of fruits and vegetables, phytochemical, bioactive compounds, nutraceutical, green extraction method, encapsulation

## Abstract

Bio-waste (i.e., peels), the by-products obtained from the processing of fruits and vegetables, represents an outstanding advance in agricultural waste valorization due to phytochemical (bioactive compounds) enrichment and the approach to a bio-circular economy and agronomic systems free of hazardous pesticides (soil remediation). These alternatives, which are environmentally friendly and sustainable, are greatly relevant to food and nutraceuticals based on bioactive compounds extracted mostly from peels. Bioactive compounds are defined as natural chemical compounds that have a positive influence on human health. They can aid in the prevention of chronic disease (cancer and degenerative, intestinal bowel and cardiovascular disease) and other types of disease. The bioactive compounds with these properties belong to the family of polyphenol compounds, which include flavonoids (i.e., flavones, flavanones, and anthocyanins), non-flavonoids (phenolic acids, stilbenes, lignin, coumarins, and tannins), and terpenes (carotenoids, lycopene, phytosterols, and monoterpenes). The extraction of these compounds from the peels of fruits and vegetables has gained increasing interest as a sustainable technology because of the use of safety solvents. Another important issue to highlight is the enormous potential of bioactive compounds, as mentioned above, in the biotechnology of these compounds, particularly in terms of the development of a delivery system targeting the site of action.

## 1. Bio-Waste (i.e., Peels) as a Phytochemical Enrichment Source of Bioactive Compounds

**Figure 1:**
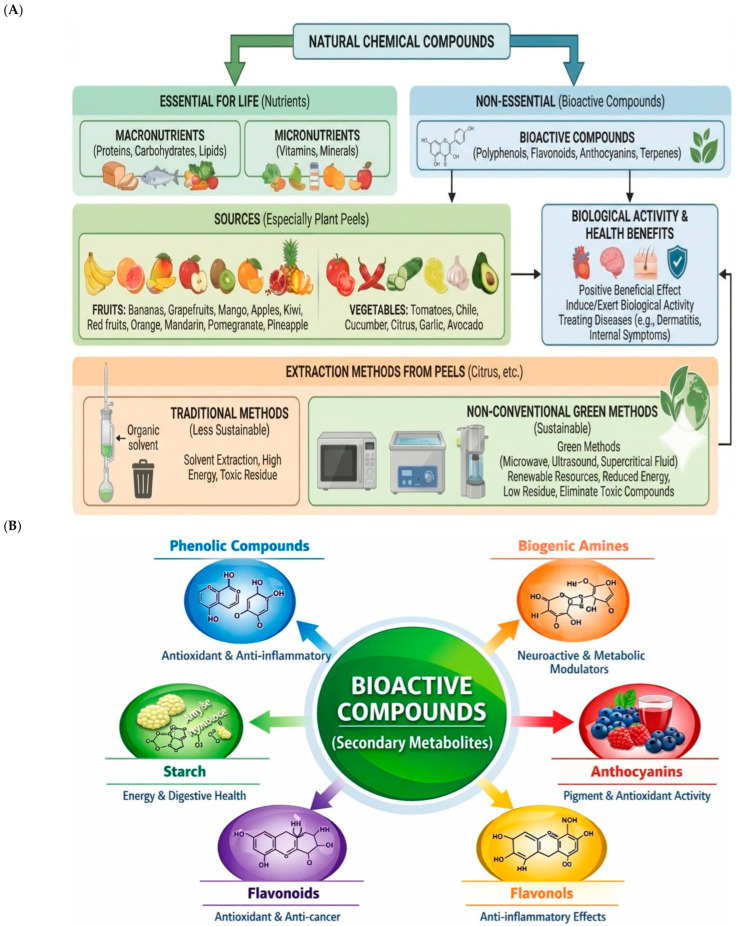
The peel derived bioactive compounds from fruits and vegetables as an emerging recognizable source of bioactive compounds with potential health benefits. (**A**) The bioactive compounds are chemical compounds, commonly found in nature as essential (for life) and non-essential. In the first case are the macronutrients (proteins, carbohydrates, lipids) and micronutrients (vitamins and minerals). In the second case are the bioactive compounds (polyphenols, flavonoids, anthocyanins, and terpenes), able to induce or exert a biological activity, extracted from different parts of the plants, especially from the peel of fruits (bananas, grapefruits, mango, apples, kiwi, red fruits, orange, mandarin, pomegranate, and pineapple) and the vegetables (tomatoes, chili, cucumber, citrus, garlic, and avocado). The extraction methods to obtain bioactive compounds from the peels of fruits, and vegetables are now non-conventional green methods because they use renewable resources, reduce energy consumption, exhibit low residue, and eliminate toxic compounds. (**B**) Bioactive compounds are able to induce or exert a biological activity, in general causing a positive beneficial effect on human health. For example, they can be used for the treatment of several diseases including dermatitis, or more internal symptoms of any disease.

Contemporary research has shifted from considering legume shells and residues as mere agricultural waste to defining them as phytonutrient-dense matrices [1,2,3,4]. The current theoretical approach is the circular bio-economy, which proposes the valorization of these by-products to reduce environmental impact and, simultaneously, extract secondary metabolites with high therapeutic value. Plant husks act as the first line of defense against biotic and abiotic stressors, resulting in a significantly greater accumulation of bioactive compounds (especially polyphenols and terpenes) compared to the edible parts or pulp [1,3,4,5]. Over the past decade, the management of agro-industrial waste has shifted from a logistical approach focused on disposal to a bio-refinery strategy. Fruit and vegetable peels, as well as legume husks, represent between 20% and 50% of the total product weight, and recent research confirms that these matrices possess phytochemical densities up to 10:1 higher than edible pulp [4,5,6]. These phytochemicals (secondary metabolites) are substances of pharmacological importance and can be found in plant-based foods and beverages [7,8], which can be extracted from different parts of herbal and medicinal plants, including the stem, leaf, flower, sap, inner trunk, root, and inner core [9,10,11]. The peel-derived bioactive compounds, defined as natural chemical compounds that promote benefits for human health [12,13,14] (Figure 1A), are commonly found in fruits, vegetables, legumes, whole grains, and nuts. The peel of fruits like bananas, apples, peaches, grapefruits, plums, and pears, and vegetables (lemon, tomato, garlic, onion, potatoes, and beans) bring enormous health benefits. As mentioned above, they can have anti-diabetic, antihypertensive, anti-inflammatory, antimicrobial, antioxidant, and antiviral effects [2,3,9,10,11]. They also possess other properties, such as neuroprotection, stimulation of the immune system, cell detoxification, cholesterol synthesis, anticonvulsant activity, and, importantly, blood pressure reduction [10,11,15]. There are two types of bioactive compounds: those that are essential for life, which include proteins, vitamins, and carbohydrates, and those that are non-essential for life, such as polyphenols (i.e., flavonoids, flavones, flavanones, and anthocyanins), non-flavonoids (phenolic acids, stilbenes, lignin, and tannins), and terpenes (β-carotene, lycopene, and phytosterol) [9,10,11,15].

Nutraceuticals (nutrition + pharmaceutical) based on the properties of bioactive compounds (phenolic compounds) obtained from seeds, peels, and fruit juice can also show potential beneficial neuroprotective effects in Alzheimer’s disease (AD) [16], as can those obtained from the peels and kernels of mango (*Mangifera indica* L.), which possess antimicrobial, anti-diabetic, anti-inflammatory, and anti-carcinogenic properties [15,16]. To obtain the maximum benefits for human health and the relevance in food industry, it is a priority to continuous monitoring and surveillance the occurrence of pesticides hazard in minor crops, to strengthen regulatory measures to warrant safety in these crops and therefore in further processing and development of nutraceutical based on the bioactive compounds extracted from the peels of fruits and vegetables. The use of hazardous pesticides is being used as a type of agriculture practice to control insect’s plague. Representing thus a risk for animal, human, and environmental health [17,18,19,20,21]. Hazardous pesticides approved or not authorized have an effect especially for children, who are highly susceptible to the negative effects in health of not approved pesticides [22,23,24,25,26,27,28,29]. The ingestion or consumption of dietary fruits and vegetables with low, moderate and high occurrence of pesticides can contribute to the development of chronic disease by affecting the gut microbiome, and several pathways of the lipid, enzyme, vitamins and xenobiotics metabolism [18,20]. Among the pesticides that have been detected in fruits and vegetables sing gas chromatography-mass spectrometry (GC-MS) or metabolomics [18] are difenoconazole, acetamiprid, carbendazim, procymidone, emamectin benzoate, lambda-cyhalothrin, cypermethrin, and dimethomorph [19].

It is also pivotal to optimize the technology to detect and identified them in the bio wastes, which possess a high concentration of bio actives, essential (vitamins, carbohydrates, and fiber micronutrients) [17,30,31,32], and not essential (polyphenols, flavonoids, flavones, anthocyanins, quercetin), rendering them suitable for circular economy, and precision farming [33], favoring lowering environmental effects and offer the enormous alternative to prevent and reuse recovered natural by-products. In other words, this aforementioned strategy rendering thus into novel raw resources [34,35], novel products with benefits for health (nutraceutical) and the food industry, thus adding a major value by the use of sustainable technology to extract nutritive components [36]. Therefore, nowadays, there is awareness of the potential of the by-products of fruit and vegetable processing (skin, pomace, seeds), since they are enriched with the bioactive compounds: phytochemicals, antioxidants, and nutrients [33,34,35]. Through the use of the bio wastes (peels of fruits and vegetables), and/or the by-products, which are obtained from the processing of the fruits and vegetables, the peels can become as an emergent recognizable source of phytochemicals enrichment and can potentiate agricultural waste valorization and agronomic systems (precision farming), thus favoring safety bioactive compounds for nutraceutical purposes (biomedical applications) [4,5,7,8,9,10,11,12,13,14].

## 2. Composition and Mechanism of Action of the Bioactive Compounds Extracted from the Peel of Fruits, Vegetables

Bioactive compounds occur naturally in plants: fruits, vegetables, legumes, whole grains, nuts, seeds, and spices [7,8], such as polyphenols compounds (flavonoids, anthocyanins, isoflavonoids), non-flavonoids (tannins, phlobatannins, stilbenes, lignans), alkaloids, glycosides, and terpenoids (carotenoids, phytosterols) [1,2,3,4,11,35]. Polyphenols [non-flavonoids (phenolic acid)] and terpenoids (carotenoids) are the predominant phytochemicals present in fruits and vegetables and are related to human health [37] (Figure 1A,B). On specifically referring to banana, *Musa* spp. is among the most cited chemical compositions in either the peel or the pulp is the carotenoids, phenolic compounds, and biogenic amines. Due to this composition, peel and pulp are considered natural sources of antioxidants and provitamin A [5,35,37]. Moreover, the peel-derived bioactive compounds from banana containing the following bioactive components: ferulic acid, protocatechualdehyde, 2-pentanone, 4-epitetracyclenone, cycloeucalenol acetate, and chlorogenic acid [5,35,37]. The peels and kernels of mango (*Mangifera indica* L.) are the source of bioactive compounds, such as phenolic compounds (flavonoids and non-flavonoids), biogenic amines, and other types of compounds, including starch. While the mango peel derived bioactive compounds in the mango peel include protocatechuic acids, Mangifera, and β-carotene [16,38,39,40,41,42]. The composition of yuzu, a citrus fruit from Northeast Asia, includes vitamin C, malic, citric acids, and four flavanones (naringin, hesperidin, naringenin, and hesperidin) [36]. The citrus peel is composed of phenolic compounds (naringin, hesperidin) and other types of bioactive compounds (terpenoids, carotenoids; essential oils, limonene, dietary fibers) [35,36]. The onion (*Allium cepa* L.) peel (especially the red variety) is the richest source of two flavonoids quercetin (aglycone form), which facilitates its rapid absorption in the organism [10,43,44], and anthocyanins that have been identified in significant concentrations in onions, especially present in red and yellow onion by-products [45,46,47,48,49,50]. The garlic peel derived bioactive compounds are mainly the tannins (gallic acid, ferulic acid, and catechin) [51,52].

### The Mechanism of Action of the Bioactive Compounds Extracted from Fruits and Vegetables

The peel-derived bioactive compounds from the fruits and vegetables, exert beneficial effects in the human health. The mechanism of action involves the antioxidant signaling pathways, activating the body’s endogenous defense systems rather than just neutralizing oxidants directly. Indeed, for both cardiovascular and neurodegenerative health, the transition from simple antioxidant activity to complex cell signaling modulation is what defines the therapeutic potential of polyphenols and terpenes (Figure 1B, Table 1 and Table 2) phenolic compounds, act in the prevention of cardiovascular disease, cancer, diabetes, and obesity [53,54,55,56,57,58] via oxidative stress and chronic inflammation mechanism. Many polyphenols in the shells act as mild stressors, inducing a gentle endogenous antioxidant response that primes cells against further damage [6]. They act as “next-generation prebiotics,” increasing the abundance of *Akkermansia muciniphila*, a bacterium associated with metabolic health [56]. Unlike isolated supplements, the compounds in peels interact synergistically with dietary fiber, enhancing its bioavailability and biological effect. Fiber-bound polyphenols in the peels of legumes and fruits are not absorbed in the small intestine; they reach the colon, where they serve as a substrate for beneficial bacteria, producing short-chain fatty acids [56,59] (Figure 1B). The bioactive compounds (biogenic amines, dopamine) extracted from banana peels can be used for wound healing or to prevent several illnesses, such as depression and Parkinson’s disease [53], and in chronic diseases, for example, against different types of cancer (i.e., breast, cervical, colorectal, esophageal, hepatic, oral, and prostate, skin) [2,3,9], in infectious disease, as antimicrobial, and anti-parasitic (leishmanicidal, helminthic) [60,61], and as therapeutic agents, hypolipidemic, and hypoglycemic [62,63,64,65]. These properties have been reported for two main parthenocarpic species of banana, *Musa accuminanta Colla* and *Musa balbisiana Colla* [2,10,11,15]. The peel of pomegranate (PF) is a source of bioactive compounds, such as polyphenol compounds, that act as multifunctional agents, as antioxidants, anti-aging, anti-inflammatory, anti-tumor, anti-obesity, and anti-diabetic in cardiovascular disease [9,55]. They have also shown neuroprotective properties and can lower cholesterol. *Citrus* peels are rich in bioactive phenolic compounds with various health effects, including antioxidant, anti-obesity [66], anti-inflammatory, antihypertensive, antihypercholesterolemic [62,63,66], antimicrobial [67,68], antidiabetic [65,69], and anticarcinogenic properties [14,70]. The photosynthetic pigments in citrus and their derivatives possess therapeutic properties, that includes antioxidant, ant mutagenic, ant genotoxic, anti-cancer, and anti-obesogenic activities [64,68] (Table 1 and Table 2).

**Table 1:** The properties of the Peels of fruits, and vegetables: benefits, composition, extraction methods and reported concentrations/dosages.

N°	Fruit/Vegetable	Peel Used?	Benefits and Role in Health	Main Chemical Composition	Extraction Methods	Reported Concentrations/ Dosages	Reference
1	Pomegranate	Yes, exclusively peel	Antioxidant, anti-inflammatory, anticancer, cardioprotective, antimicrobial	Punicalagin (70% of polyphenols), ellagitannins, anthocyanins, ellagic acid, tannins	Solvent extraction (50% aqueous methanol), enzyme-assisted extraction, UAE, MAE	Extract: 1.0–5.0 mg/mL Punicalagin: 0.8–3.2 mg/g In vivo: 100–400 mg/kg	[71]
2	Apple	Yes, specific study of peel	Antioxidant, prevention of chronic diseases, cholesterol reduction	Gallocatechin, epicatechin, epigallocatechin, pectin, polyphenols, quercetin	Enzymatic extraction (cellulolytic and pectolytic), liquid extraction with methanol/ethanol	Aqueous extract: 500 mg/kg body weight Polyphenols: 2–10 mg GAE/g Concentration: 1–5 mg/mL	[72]
3	Orange	Yes, peel as main residue	Anti-inflammatory, blood sugar regulation, anticancer (squamous skin cells), cognitive improvement	Hesperidin, vitamin C, carotenoids, limonene, pectin, flavonoids	Microwave-assisted extraction (MAE), liquid extraction with methanol, supercritical extraction	Hesperidin: 50–200 mg/day Extract: 0.5–2.0 g/day Concentration: 10–50 mg/mL	[73]
4	Banana	Yes, exclusively peel	Antioxidant, neuroprotective, improves brain function, weight control	Dopamine, L-DOPA, catecholamines, polyphenols (232 mg/100 g), flavonoids, carotenoids	Liquid extraction with methanol, ethanol, acetone, water acidified with HCl	Extract: 100–500 mg/kg Polyphenols: 200–250 mg/100g Concentration: 5–20 mg/mL	[74]
5	Mandarin	Yes, focused on peel	Antioxidant, hydroxyl radical elimination, anti-inflammatory properties	Polyphenols (37,793.37 μg/g), phenolic acids, flavanones, hesperidin, nobiletin	Microwave-assisted extraction (1–120 °C/999 s), ultrasound-assisted extraction (30–40 °C)	Extract: 200–600 mg/kg Polyphenols: 30–40 mg/g Hesperidin: 25–100 mg/day	[75]
6	Kiwi	Yes, peel and pulp (comparative)	Antioxidant, protection against oxidative stress, anti-inflammatory	Polyphenols (51.2 mg GAE/g), vitamin C, actinidin, flavonoids, phenolic acids	Subcritical water extraction (160 °C/20 min), conventional liquid extraction	Extract: 100–300 mg/kg Polyphenols: 40–60 mg GAE/g Concentration: 2–10 mg/mL	[76]
7	Mango	Yes, exclusively peel	Antimicrobial, antidiabetic, anti-inflammatory, anticancer, body fat reduction	Phenolic acid, Mangifera, beta-carotene, vitamin C, carotenoids, pectin (90–110 mg/g)	Liquid extraction with acetone, ultrasound-assisted extraction, maceration	Extract: 150–500 mg/kg Mangifera: 10–50 mg/day Concentration: 5–25 mg/mL	[77]
8	Melon	Yes, only peel	Antioxidant, cardiovascular health improvement, hydration	Polyphenols (0.69–2.96 mg GAE/g), flavonoids, carotenoids, cucurbitacin	Liquid extraction with methanol (25 °C/15 min), hydroalcoholic extraction	Extract: 200–400 mg/kg Polyphenols: 1–3 mg GAE/g Concentration: 1–5 mg/mL	[78]
9	Tomato	Yes, peel as main study object	Antioxidant, anticancer, cardiovascular protection, improvement of meat products	Lycopene, beta-carotene, polyphenols, flavonoids, chlorogenic acid, quercetin	Extraction with organic solvents, ultrasound-assisted extraction, maceration	Extract: 100–400 mg/kg Lycopene: 5–30 mg/day Concentration: 10–50 mg/mL	[79]
10	Potato	Yes, specifically peel	Antioxidant, anti-inflammatory, anticancer, gut health improvement	Phenolic acids (chlorogenic acid, caffeic acid), glycoalkaloids, anthocyanins (in purple varieties)	Hydroalcoholic extraction, methanol extraction, enzyme-assisted extraction	Extract: 100–300 mg/kg Chlorogenic acid: 50–200 mg/day Concentration: 5–15 mg/mL	[54]
11	Onion	Yes, outer layer (peel)	Antidiabetic, antioxidant, anti-inflammatory, cardiovascular function improvement, antimicrobial	Quercetin and derivatives, flavonoids, sulfur compounds, anthocyanins (red onions), kaempferol	Ultrasound-assisted extraction, maceration with ethanol, subcritical water extraction	Extract: 200–600 mg/kg Quercetin: 50–500 mg/day Concentration: 10–30 mg/mL	[80]
12	Garlic	Yes, outer peel (pericarp)	Antimicrobial, antioxidant, anti-inflammatory, immune system improvement	Phenolic compounds, flavonoids, organosulfur compounds (allicin), saponins	Extraction with different solvents, maceration, ultrasound-assisted extraction	Extract: 100–400 mg/kg Allicin equivalent: 300–1200 mg/day Concentration: 5–20 mg/mL	[81]
13	Papaya	Yes, specific focus on peel	Antioxidant, anti-inflammatory, improves digestion, prevention of chronic diseases	β-carotenes, flavonoids, polyphenols, papain, vitamin C, lycopene	Liquid extraction, maceration with polar solvents, drying and grinding	Extract: 150–400 mg/kg Polyphenols: 10–30 mg/g Concentration: 5–15 mg/mL	[82]
14	Carrot	Yes, peel as byproduct	Antioxidant, vision protection, anticancer, skin improvement	Carotenoids (α and β-carotene), polyphenols, dietary fiber, anthocyanins (purple carrots)	Extraction with organic solvents, maceration, ultrasound-assisted extraction	Extract: 100–500 mg/kg β–carotene: 6–15 mg/day Concentration: 5–20 mg/mL	[82]
15	Eggplant	Yes, exclusively peel	Antioxidant, anti-inflammatory, cholesterol reduction, neuroprotective	Anthocyanins (Nasuni), polyphenols, chlorogenic acid, delphinidin, flavonoids	Extraction with polar solvents, maceration, hydroalcoholic extraction	Extract: 100–400 mg/kg Nasuni: 20–100 mg/day Concentration: 5–15 mg/mL	[82]
16	Lime	Yes, peel as main study object	Antioxidant, antimicrobial, anti-inflammatory, immune system improvement	Flavonoids (222.3–282.5 mg EQ/100 g), hesperidin, limonene, vitamin C, polyphenols	Extraction with organic solvents, cold press extraction, maceration	Extract: 200–500 mg/kg Flavonoids: 100–300 mg/day Concentration: 10–25 mg/mL	[82]
17	Watermelon	Yes, white and green peel	Antioxidant, anti-inflammatory, hydration, cardiovascular improvement	Lycopene, citrulline, carotenoids, flavonoids, vitamin C	Extraction with solvents, lyophilization, ultrasound-assisted extraction	Extract: 200–600 mg/kg Citrulline: 1–6 g/day Concentration: 5–20 mg/mL	[83]
18	Peach	Yes, peel compared with pulp	Antioxidant, anticancer, anti-inflammatory, skin protection, antimicrobial	Chlorogenic acid, neochlorogenic acid, catechin, epicatechin, procyanidins, anthocyanins	Ultrasound-assisted extraction, pressurized liquid extraction, maceration with methanol	Extract: 100–400 mg/kg Polyphenols: 15–40 mg/g Concentration: 5–20 mg/mL	[84]
19	Pear	Yes, specifically peel	Antioxidant, digestion improvement, glycemic control, cholesterol reduction, anti-obesity	Arbutin, chlorogenic acid, catechin, epicatechin, procyanidin B2, quercetin glycosides	Ultrasound-assisted extraction, maceration with ethanol, supercritical fluid extraction	Extract: 150–500 mg/kg Arbutin: 50–200 mg/day Concentration: 5–20 mg/mL	[85]
20	Grape	Yes, skin (peel)	Antioxidant, cardioprotective, neuroprotective, longevity increase, anti-inflammatory	Resveratrol, procyanidin B1, anthocyanins, quercetin, catechins, tannins	Extraction with alcoholic solvents, maceration, ultrasound-assisted extraction	Extract: 100–500 mg/kg Resveratrol: 10–500 mg/day Concentration: 10–50 mg/mL	[86]
21	Persimmon	Yes, peel only	Antioxidant, anti-inflammatory, cardiovascular improvement, antidiabetic, anticancer	Proanthocyanins, catechins, gallic acid, carotenoids (β-cryptoxanthin), tannins (higher in peel than pulp)	Ultrasound-assisted extraction, maceration with methanol, freeze-drying	Extract: 100–400 mg/kg Proanthocyanins: 50–200 mg/day Concentration: 5–15 mg/mL	[87]
22	Spinach	Yes, outer leaves considered as peel	Antioxidant, anti-inflammatory, bone health improvement, eye protection	Polyphenols, flavonoids, lutein, zeaxanthin, beta-carotene, vitamin K	Hydro methanol extraction, hydro ethanol extraction, green extraction	Extract: 200–600 mg/kg Lutein: 6–20 mg/day Concentration: 10–30 mg/mL	[73]
23	Passion Fruit	Yes, specifically peel	Antioxidant, antihypertensive, anti-anxiety, hypoglycemic, high-quality dietary fiber source	Pectin (15–30%), flavonoids (including luteolin, quercetin), phenolic compounds, piceatannol, carotenoids	Hot water extraction, acid extraction, microwave-assisted extraction, enzymatic extraction	Extract: 100–500 mg/kg Pectin: 5–15 g/day Concentration: 10–30 mg/mL	[88]
24	Pumpkin	Yes, outer peel	Antioxidant, immunomodulatory, antidiabetic, anti-obesity, antimicrobial, wound healing	Carotenoids (β-carotene, lutein), phenolic compounds, tocopherols, phytosterols, pectin, cucurbitacin	Green extraction, pulsed electric field extraction, enzyme-assisted extraction	Extract: 150–500 mg/kg β–carotene: 5–15 mg/day Concentration: 5–20 mg/mL	[89]
25	Cantaloupe Melon	Yes, exclusively peel	Antioxidant, antimicrobial, anti-inflammatory, anti-obesity, improved gut health	Polyphenols (gallic acid, chlorogenic acid, caffeic acid), flavonoids, carotenoids, dietary fiber (60–70%)	Ultrasound-assisted extraction, microwave-assisted extraction, freeze-drying	Extract: 200–500 mg/kg Polyphenols: 1–3 mg GAE/g Concentration: 5–15 mg/mL	[90]
26	Asparagus	Yes, outer part discarded as by-product	Prebiotic, antioxidant, gut microbiota modulation, anti-inflammatory	Inulin, dietary fiber, low and high molecular weight polyphenols, saponins	Hot-water extraction, ultrasound-assisted extraction, enzyme-assisted extraction	Extract: 100–400 mg/kg Inulin: 5–15 g/day Concentration: 5–20 mg/mL	[91]
27	Turmeric	Yes, outer peel of the rhizome	Anti-inflammatory, antioxidant, anticancer, neuroprotective, antimicrobial	Curcumin, polyphenols, flavonoids, dietary fiber (soluble and insoluble), essential oils	Extraction with ethanol, ultrasound-assisted extraction, supercritical extraction	Extract: 500–2000 mg/day Curcumin: 500–2000 mg/day Concentration: 20–100 mg/mL	[92]
28	Pineapple	Yes, peel as main residue	Antioxidant, anti-inflammatory, improves digestion, antimicrobial	Bromelain, polyphenols, vitamin C, dietary fiber, phenolic acids	Enzymatic extraction, extraction with water, maceration with ethanol	Extract: 200–600 mg/kg Bromelain: 200–800 mg/day Concentration: 10–30 mg/mL	[93]
29	Lemon	Yes, exclusively peel	Antimicrobial, antioxidant, anti-inflammatory, anticancer, neuroprotective, food preservative	D-limonene (68.5%), γ-terpinene, β-pinene, citrus, flavonoids (hesperidin, naringin), phenolic acids, pectin	Hydro distillation, cold-pressing, supercritical CO_2_ extraction, microwave-assisted extraction	Essential oil: 0.1–1% in foods Extract: 200–500 mg/kg D–limonene: 100–500 mg/day	[94]
30	Jackfruit	Yes, outer peel	Antioxidant, antimicrobial, anti-inflammatory, waste reduction, agricultural applications	Flavonoids, polyphenols, steroids, tannins, saponins, terpenoids and triterpenoids	Solvent extraction, subcritical water extraction, drying and grinding	Extract: 100–400 mg/kg Polyphenols: 10–30 mg/g Concentration: 5–20 mg/mL	[95]

**The polymethoxylated flavonoids (PMF)** present in the citrus peel of hesperidin, naringin [15,56,65,69], and act by inhibiting the HMG-CoA reductase enzyme, similar to statins, but with superior safety profiles or by modulating redox and pro-inflammatory signaling pathways [63,64]. Pre-clinical and clinical studies [4,62,63,64,65,66] have assessed that supplementation with citrus peel extracts reduces LDL cholesterol and improves insulin sensitivity [96] (Table 2). The primary mechanistic goal in cardiovascular health is maintaining vascular homeostasis and preventing the oxidation of low-density lipoproteins (LDL). In this sense, polyphenols (especially anthocyanins and flavanols) stimulate eNOS (endothelial Nitric Oxide Synthase) by triggering the PI3K/Akt signaling pathway, which increases the production of Nitric Oxide (NO). NO is a potent vasodilator that relaxes smooth muscle cells, reducing blood pressure and improving arterial stiffness [60,97,98,99,100,101]. In the Inhibition of protein aggregation (proteostasis), polyphenols, like Epigallocatechin gallate (EGCG), and curcumin directly interfere with the misfolding of proteins. Polyphenol bind to amyloid-beta (Aβ) monomers or tau proteins, preventing them from forming the toxic oligomers and plaques that disrupt neuronal communication [10,102]. In neurotrophic support, polyphenols (some flavonoids) increase the expression of BDNF (Brain-Derived Neurotrophic Factor), promoting the survival and growth of new neurons. Furthermore, onion peel extracts have been highlighted with numerous properties of the anticancer, antibacterial, anti-obesity, neuroprotective, cardioprotective, antidiabetic, and erectile dysfunction activities [16,49,68].

**Table 2:** Dosage reported of fruits and vegetables to obtain measurable physiological effects in humans or advanced animal models.

Vegetable Matrix	Bioactive Compound	Reported Dose	Therapeutic Application	Reference
Pomegranate peel	Punicalagins Ellagic acid	500–100 mg/day	Reduction in arterial inflammation	[4]
Orange peel	Polymethoxyflavones	250–500 mg/kg	Hypolipidemic and anti-obesity effect	[5]
Apple peel	Ursolic acid Quercetin	3–5 g/day (Dehydrated powder)	Prevention of sarcopenia and redox stress	[10]
Grape peel	Resveratrol Pro anthocyanidins	200–400 mg/day	Neuroprotection and anti-aging	[56]
Mango peel	Mangifera	200 mg/day	Modulation of metabolic syndrome	[96]
Lemon peel	Eritriocin	400–800 mg/day	Reduction in uric acid and gout	[8]
Tomate peel	Lycopene	15–30 mg/day	Protection against prostate cancer	[103]
Beans peel	Anthocyanins Catechins	100 mg/kg weight	Postprandial glucose control	[102]
Potatoes peel	Chlorogenic acid	100 mg/day	Prebiotic action and colon health	[104]

**Figure 2:**
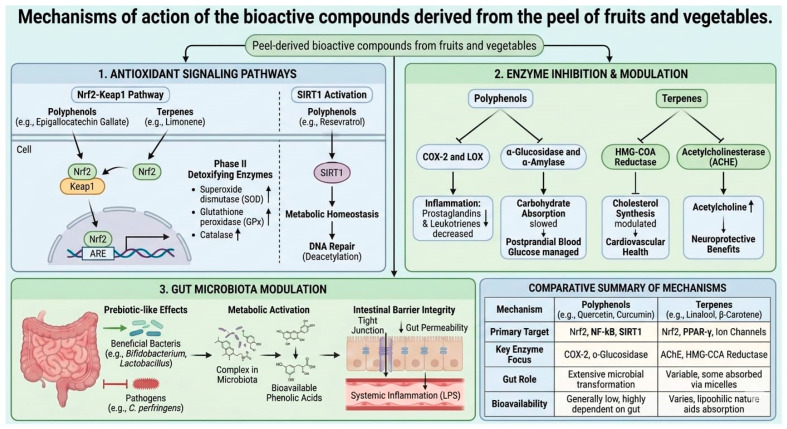
Mechanisms of action of the bioactive compounds derived from the peel of fruits and vegetables. The mechanism of action of the peel-derived compounds (Polyphenols, and Terpenes is carried out through four major routes: 1. Antioxidant signaling pathways, targeting the signalization pathways Nrf2, NF-κB, SIRT1, Nrf2, PPAR-γ, trigger the release of the transcription factor Nrf2, which migrates to the nucleus and binds to the antioxidant Response Element (ARE). This induces the expression of phase II detoxifying enzymes: Superoxide dismutase (SOD), Glutathione peroxidase (GPx), and ion channels. 2. Enzyme Inhibition and modulation. Polyphenols and terpenes act as inhibitors of enzymes involved in chronic inflammation and metabolic dysfunction, such as COX-2, α-Glucosidase, AChE, and HMG-CoA Reductase. 3. Gut microbiota modulation. 3. Polyphenols and terpenes promote the growth of “beneficial” bacteria (Prebiotic-like Effects). 4. Cardiovascular and neurodegenerative disease. Polyphenols and terpenes targeting the endothelium (eNOS activation) and Microglia (NF-κB inhibition). This caused vasodilation, Lower Blood Pressure, and Reduced Neuroinflammation, leading to the Prevention of low-density lipids oxidation and Protein Aggregation (Plaques).

**Terpenes such** as-Lycopene, extracted from the skin of tomatoes is involved in the specific transport proteins that improve prostate health [103]. Consuming lycopene from the skin is correlated with a decreased risk of prostate cancer and protection against UV-induced skin damage [54]. The terpene’s role (like oleanolic acid) as bioactive compounds in this disease are the modulation of the renin-angiotensin system, acting as natural inhibitors of ACE (Angiotensin-Converting Enzyme). In the lipid Peroxidation, and Atherosclerosis [60,97,98,99]. Terpenes act through a mechanism of “Antioxidant Signaling” mentioned previously (Nrf2) upregulates paraoxonase 1 (PON1), which is an enzyme associated with HDL (“good” cholesterol) that prevents the oxidation of LDL. Since oxidized LDL is a primary trigger for plaque formation (atherogenesis), these compounds act as a preventive shield for the arterial wall. In neurodegenerative health, proteostasis and neuroinflammation like Alzheimer’s or Parkinson’s, the mechanisms shift toward clearing “molecular trash” and calming the brain’s immune response [55]. In Autophagy Induction, many terpenes (like linalool) stimulate autophagy via the AMPK pathway—essentially a cellular “recycling” process that degrades damaged proteins before they become toxic. Furthermore, in Microglial Modulation (anti-inflammation), both polyphenols and sesquiterpenes inhibit the NF-κβ and MAPK pathways in microglia [1,2,3,4]. This drastically reduces the release of pro-inflammatory cytokines like TNF-α and IL-1β, which otherwise lead to neuronal death, causing chronic neurodegeneration driven by “overactive” microglia (the brain’s immune cells) [98,100,101]. On the other hand, bioactive compounds can share mechanism of action (Table 2). Thus, for example, -Polyphenols (like epigallocatechin gallate) and Terpenes (like limonene), trigger the release of the transcription factor Nrf2, which migrates to the nucleus and binds to the antioxidant Response Element (ARE). This induces the expression of phase II detoxifying enzymes: Superoxide dismutase (SOD), Glutathione peroxidase (GPx), Catalase and SIRT1 Activation (Figure 2). Polyphenols (notably resveratrol) activate Sirtulin 1, a deacetylase that regulates metabolic homeostasis and DNA repair, mimicking the effects of caloric restriction [100,101]. In addition, polyphenols and terpenes act as natural inhibitors of key enzymes involved in chronic inflammation and metabolic dysfunction. In particular, polyphenols, i.e., flavonoids, inhibit Cyclooxygenase-2 and Lipoxygenase, reducing the production of pro-inflammatory prostaglandins and leukotrienes, inhibit the digestive enzymes α-Glucosidase and α-Amylase, and slow down carbohydrate absorption, helping manage postprandial blood glucose levels [102]. Terpenes, certain monoterpenes, can modulate the rate-limiting enzyme (HMG-CoA Reductase) in cholesterol synthesis, contributing to cardiovascular health [63,64,65,69]. Terpenes, like 1, 8-cineole (eucalyptol), can inhibit AChE, which increases the availability of acetylcholine in the brain, offering potential neuroprotective benefits [5,13]—polyphenols and certain di terpenes promote the growth of “beneficial” bacteria (prebiotic-like effects), such as Bifidobacterium and *Lactobacillus* while inhibiting pathogens like *Clostridium perfringens.* Most polyphenols reach the colon unabsorbed. Microbiota break down complex structures (like pro anthocyanins) into smaller, more bioavailable phenolic acids that can then enter the bloodstream to exert systemic effects. In addition, these compounds strengthen “Tight Junction” proteins, reducing gut permeability (often called “leaky gut”) and preventing systemic inflammation caused by endotoxins like LP [4,12,15,16,56,102,104] (Figure 2; Table 1 and Table 2).

## 3. Methods for Bioactive Compounds Extraction from the Peel of Fruits, and Vegetables

The methods that are most environmentally friendly for the extraction of the bioactive compounds from the peel of fruits and vegetables are the modern non-conventional green methods, since the conventional methods suffer from significant drawbacks such as long extraction times (hours to days), high solvent and energy consumption, potential thermal degradation of sensitive compounds, and environmental concerns due to toxic solvent residues [103,105,106,107]. Despite this, solvent extraction (maceration), Soxhlet extraction, and hydro distillation are characterized by simple, well-established protocols and cost-effective equipment, and the phenolic can be recovered from the fruit peels by conventional extraction methods; no extractable phenolic remaining in the residues must be released from the cell matrix first by hydrolysis with acid, alkali, or enzymes [105,107]. The green extraction techniques—including ultrasound-assisted extraction (UAE), microwave-assisted extraction (MAE), enzyme-assisted extraction (EAE), supercritical fluid extraction (SFE), subcritical water extraction (SWE), and pulsed electric field extraction (PEF)—offer substantial improvements in sustainability and efficiency [108,109,110,111,112]. These modern technologies are non-conventional, dramatically reduce extraction times (from hours to minutes or even seconds), lower solvent consumption by 50–90%, operate at lower temperatures to preserve thermo labile compounds, and minimize environmental impact through the use of green solvents like water, ethanol, or supercritical CO_2_ [113,114,115,116]. The green methods take advantage of by-products, use renewable resources, reduce energy consumption, exhibit low residue, and eliminate toxic compounds. In practice, petroleum-derived solvents can be replaced with water, ethanol, agro-solvents, or deep eutectic solvents (DES) [40,103,106,113].

The extraction techniques described in Table 1 are characterized by their efficiency and benefits. For example, the microwave-assisted extraction (MAE) uses microwaves to heat the material and facilitate the release of compounds [103,107,108,109,110,113,116]. It is also fast and efficient, with lower solvent and energy consumption. It is used for citrus fruits such as tangerines and oranges. (2) The supercritical fluid extraction uses supercritical CO_2_ to extract compounds without leaving toxic residues, offering high selectivity and preservation of sensitive compounds. It is an advanced and clean technique used in citrus fruits and pears. (3) The enzymatic extraction method uses enzymes to break down the cell structure and release compounds. It is specific and can improve yield, although it can be more expensive. (4) The ultrasound-assisted extraction (UAE) uses ultrasonic waves to break down cell walls and release bioactive compounds [109,110,111,112]. It is fast, efficient, reduces solvent use, and preserves the quality of the compounds. It is used to extract polyphenols, flavonoids, and antioxidants from various peels, such as oranges, mangoes, tomatoes, and onions, among others. However, the most efficient, fast, and environmentally friendly technique for extracting bioactive compounds from fruit and vegetable peels is the ultrasound-assisted extraction (UAE) [109,110] (Table 1). Ultrasound-assisted, microwave-assisted, enzyme-assisted, CO_2_-assisted, high-intensity pulsed electric field (Table 1). Using these green methods, the extraction of bioactive compounds of citrus fruits such as lemon, grapefruit, mandarin, orange, and pomelo has been carried out [113]. However, one of the disadvantages of the green techniques is that they require higher initial equipment investments, specialized operational expertise, and careful optimization for specific applications. Therefore, the shift toward green extraction methods represents a significant advancement in balancing extraction efficiency, product quality, environmental responsibility, and operational sustainability in industrial and research settings. It is extremely important to peel washing before processing for the extraction of the bioactive compounds (polyphenolic acids, flavonoids), which can be analyzed using chromatographic methodologies (i.e., HLPC-DAD) [114], such as gas chromatography coupled to mass spectrometry for the detection of contaminants [115].

## 4. Biotechnology of the Peel-Derived Bioactive Compounds of Fruits and Vegetables

The extreme process or medium conditions degrade phenolic compounds and their bioactivity. To overcome enzymatic degradation of the compounds, encapsulation can be applied to improve their stability, solubility, and bioactivity of the peel derived bioactive compounds from fruits and vegetables [2,3,4,103,114,115,116]. Another aspect that is important to consider in the extraction of bioactive compounds from the peels is to harness the by-products containing several essential biologically active molecules (fatty acids, proteins, carbohydrates, dietary fibers, vitamins, and minerals) [2,3,4,103,114,115,116]. Briefly, one of the most important requisites for the effective action of the bioactive compounds is that they reach the site of action to exert their biological properties as above pinpointed [4,5,6,9,10,11,12,50,55,68,114]. How can this be accomplished? One of the methods that has shown to be very effective for the delivery of bioactive compounds is encapsulation. This biotechnology allows conservation of the integrity and to reach the sites of action, improving their bio-accessibility, and it is nowadays considered a very friendly environment technology to meet the requirements of fabrication and effectiveness [117]. The bioactive compounds can be entrapped to safeguard the active ingredients. The encapsulation can be biodegradable and increase the stability and durability under field conditions of the bio stimulants. This has been shown for several bioactive compounds. For example, (-)-epigallocatechin gallate (EGCG) encapsulation and delivery has used proteins, carbohydrates, and lipids because higher biodegradability, biocompatibility, and bio functionality properties [118]. Other materials that have been used for the encapsulation of biological agents, or drug delivery systems, are the hydrogels, which provide protection against enzymatic degradation [6,119,120]. In addition, the monoaxial spraying and electro spraying are common and economical techniques used for encapsulation of nutrients and bioactive compounds for oral delivery systems to protect from degradation by gastrointestinal enzymatic system via the formation of shell-core structures [6,121]. Another alternative for the encapsulation of phenolic compounds are the natural polysaccharides, in particular the hemicellulose, whose properties are noteworthy and well suited due to their biocompatibility and biodegradation properties, making good synergy with the polyphenols [6,7,8,122].

## 5. Remarks

The bioactive compounds chemical compounds divided into essential and non-essential, commonly found in fruits, vegetables, dietary fibers, and whole grains. The peel of fruits and vegetables are enriched with bioactive compounds no essential as polyphenols and terpenes. Many of these compounds have potential benefits for human health. They act through different mechanisms of actions, with a co adjuvant role within the immunity system and the gut microbiota. Indeed, preclinical and clinical studies have shown that a diet rich in fruits and vegetables might have a synergistic effect of the bioactive compounds present acting and targeting different signalization pathways of the metabolism, immune, and neuroendocrine system, thus enhancing their enormous therapeutic value.

The methods of extraction of the bioactive compounds from the peel of fruits and vegetables is critical to obtain good yield and optimal concentration. Ultrasound-assisted extraction (UAE) has been shown as the most efficient, fast, and environmentally friendly technique for extracting bioactive compounds from different fruit and vegetable peels. An example is the case of the kiwi peel, in which the method of extraction of the bioactive compounds is based on subcritical water extraction (160 °C/20 min) and conventional liquid extraction. The yield has been good using these methods. The continuous monitoring of the occurrence of hazardous pesticides in fruits and vegetables is important, since children are the population that the most highly vulnerable to the negative effects of the toxicity of non-approved compounds in health. To aid to this problem, the agronomic systems, as precision farming, are of enormous value to obtain safe products for food industry and nutraceutical purposes, that is why the bio-waste represents phytochemical enrichment derived from peels, favoring the bio circulation economy and the agricultural waste valorization of bioactive compounds. The biotechnology of these compounds through encapsulation can allow and facilitate that they can reach the target (tissue, organ) and exert effectively their pharmacological effect.

## Data Availability

This review was based on searches and data from the PubMed database without limitation to 2025.
